# Production of genome-edited *Daphnia* for heavy metal detection by fluorescence

**DOI:** 10.1038/s41598-020-78572-z

**Published:** 2020-12-08

**Authors:** Takuto Arao, Yasuhiko Kato, Quang Dang Nong, Hiroshi Yamamoto, Haruna Watanabe, Tomoaki Matsuura, Norihisa Tatarazako, Kazune Tani, Akira Okamoto, Takeru Matsumoto, Hajime Watanabe

**Affiliations:** 1 Department of Biotechnology, Graduate School of Engineering, Osaka University, Suita, Osaka Japan; 2 Frontier Research Base of Global Young Researchers, Graduate School of Engineering, Osaka University, Suita, Japan; 3 Center for Health and Environmental Risk Research, National Institute for Environmental Studies, Tsukuba, Japan; 4 Faculty of Agriculture, Ehime University, Matsuyama, Ehime Japan; 5 Department of Environmental Science and Toxicology, Nippon Soda Co., Ltd., Kanagawa, Japan

**Keywords:** Environmental chemistry, Assay systems

## Abstract

Aquatic heavy metal pollution is a growing concern. To facilitate heavy metal monitoring in water, we developed transgenic *Daphnia* that are highly sensitive to heavy metals and respond to them rapidly. Metallothionein A, which was a metal response gene, and its promoter region was obtained from *Daphnia magna*. A chimeric gene fusing the promoter region with a green fluorescent protein (GFP) gene was integrated into *D. magna* using the TALEN technique and transgenic *Daphnia* named *D. magna* MetalloG were produced. When *D. magna* MetalloG was exposed to heavy metal solutions for 1 h, GFP expression was induced only in their midgut and hepatopancreas. The lowest concentrations of heavy metals that activated GFP expression were 1.2 µM Zn^2+^, 130 nM Cu^2+^, and 70 nM Cd^2+^. Heavy metal exposure for 24 h could lower the thresholds even further. *D. magna* MetalloG facilitates aqueous heavy metal detection and might enhance water quality monitoring.

## Introduction

Excessive amounts of heavy metals are hazardous to human and ecological health. Heavy metals are discharged from industrial and natural sources. They are major aquatic toxicants, so the concern around heavy metal pollution in water is increasing in recent years. The maximum allowable metal concentrations in environmental and drinking water are strictly regulated in many countries. For regulation of aquatic heavy metal, many monitoring methods have been developed^1^, including instrumental analyses such as flame atomic absorption spectrometry, electrothermal atomic absorption spectrometry, inductively coupled plasma optical emission spectrometry, and inductively coupled plasma mass spectrometry^2^. Nevertheless, these techniques are expensive and time-consuming; therefore, simple, rapid, and efficient methods are desired for continuous heavy metal monitoring (REF).

Biomonitoring is one of the simplest ways to detect toxicants, i.e., evaluating toxicity based on biological responses. Many organisms such as algae, zooplankton, insects, and fish have been used as biomonitors for aquatic pollutants in the past^3^. In addition to these conventional species, frogs has also been studied as a target species for heavy metals^4^. However, advances in genetic engineering have changed the classical endpoints, because gene expression change can be used as one of the endpoints. Alterations in gene expression occur before phenotypic changes appear, with the latter utilized as classical endpoints. Recently, the use of toxicogenomics based on transcription profiles has emerged^5^. It detects and explains global transcription changes induced by toxicants and it is recognized as a sensitive method; however, it is time-consuming and costly. It is informative to evaluate the expression of target genes that can be activated by toxicants. Moreover, the application of green fluorescent protein (GFP) in transgenic animals facilitates detection of gene expression changes caused by environmental changes or the presence of toxicants. In these transgenic animals, GFP is designed to be induced by the promoter of an activated gene. To monitor water quality, transgenic zebrafish and medaka have been developed^6^ to detect specific pollutants including metals^7^, aromatic carbons^8^, and estrogens^9^.

Gene expression changes in response to heavy metals have been extensively investigated. Metallothionein is one of the genes activated by heavy metal exposure^10^. It is a small, highly conserved protein found in organisms ranging from bacteria to humans^11^, and is rich in cysteine residues, the thiol groups of which bind metals. Metallothionein is, therefore, be an excellent biomarker for metal exposure^12^. Transgenic fish carrying the red fluorescent protein gene DsRed2 under the control of the metallothionein gene have been constructed^13^. Metallothionein gene Ia1 obtained from green mussels was fused to DsRed2 and introduced into zebrafish. Transgenic zebrafish responded to 4.4 μM Cd, 7.9 μM Cu, and 306 μM Zn. The use of the metallothionein gene was also reported for *Caenorhabditis elegans*^14^, whose lowest observed effect concentrations were 5 μM for Cd, 10 μM for Cu, and 250 μM for Zn. The use of transgenic animals for metal monitoring is simple; however, the detection limit is still very high.

*Daphnia magna* is a small freshwater crustacean that is used as an aquatic pollution bioindicator and model organism in ecotoxicology. As a rule, the adverse effects of aquatic pollutants have been evaluated using standard protocols such as OECD test guidelines 202 and 211 for acute and chronic toxicity testing, respectively. *Daphnia* are generally more sensitive to environmental changes than fish, and therefore it is expected that transgenic *Daphnia* could be used as an indicator of heavy metals. It has been reported that the metallothionein gene of *D. pulex* is activated by exposures to Cd^11^ and other heavy metals^15^. Therefore, the daphnid metallothionein gene might serve as a sensitive biomarker even in *Daphnia*. Recently, we successfully applied genome editing to *Daphnia*^16–19^ as well as conventional transgenic methods^15^. In the present study, we developed *D. magna* as a transgenic biomonitoring animal. Using the genome editing technique, we selected the metallothionein gene as a biomarker and visualized changes in its expression by linking it to GFP, thereby generating germ line transmitted transgenic *Daphnia.*

## Materials and methods

### Metal analysis

Test solution samples for chemical analysis were prepared in a plastic cup, separate from each test. The samples were collected at the start and the end of the exposure period to determine dissolved heavy metal concentrations. The collected samples were diluted using ultrapure water and nitric acid (final concentration of 0.13 M), spiked with an internal standard (Ga and In, the final concentration of 2 μg/L and 10 μg/L), and then analyzed using ICP-MS (Agilent 8800, Agilent Technologies, Inc.).

### *Daphnia* culture

*D. magna* (NIES clone) were obtained from the National Institute for Environmental Studies (Tsukuba, Japan). Cultures with a density of 16 individuals/L were maintained at 23 ± 1 °C under a 14 h light/10 h dark photoperiod in 5 L of Aachener Daphnien Medium (ADaM)^20^. A 0.08 mL aliquot of a 7 × 10^9^ cells/mL *Chlorella* suspension was added daily to each culture^21^.

### Heavy metal exposure

For mRNA expression analysis of MT genes, neonate *Daphnia* less than 24 h old were used, which corresponded to < 96 h after oviposition because it takes 72 h for the neonate to swim out from the brood chamber. For GFP fluorescence analysis, juvenile *D. magna MetalloG Daphnia* (7 d post-oviposition) was used. Five each of neonate or juvenile daphniids were placed in 250 ml of ADaM medium and exposed to various metal concentrations over a 24-h or 48 h period. They were incubated at 23 ± 1 °C under a 14 h light/10 h dark photoperiod. Stock solutions of ZnCl_2_, CuSO_4_, and CdCl_2_ (Nacalai Tesque; Kyoto, Japan) were added to make up the final metal concentrations. Nominal concentrations were 0.59 μM (80 μg/l), 2.9 μM (400 μg/l), and 14.7 μM (2000 μg/l) for the ZnCl_2_ exposure, 64 nM (10.2 μg/l) and 320 nM (51 μg/l) for CuSO_4_ exposure. There was no feeding during exposure to heavy metals. Exposures were performed independently in triplicate.

### Quantification of mRNA

Total RNA was extracted from five daphniids using Sepasol-RNA I solution (Nacalai Tesque; Kyoto, Japan) according to the manufacturer’s protocol. Around 0.5 μg of total RNA was generally obtained. Concentrations of RNA were determined by A260 measured by Nanodrop. One-fifth of the total RNA was used in cDNA synthesis with 250 ng of random primers (Invitrogen, Carlsbad, CA, USA) and 200U of SuperScript III Reverse Transcriptase (Invitrogen, Carlsbad, CA, USA). Quantitative polymerase chain reaction (qPCR) was performed with SYBR GreenER qPCR Supermix Universal (Invitrogen, Carlsbad, CA, USA) using the Mx3005P real-time (RT)-PCR system (Agilent Technologies, Santa Clara, CA, USA). PCR amplifications were performed with 200 nM primers in triplicate using the following conditions: 2 min at 50 °C and 10 min at 95 °C, followed by a total of 40 two-temperature cycles (15 s at 95 °C and 1 min at 60 °C) in a volume of 20 μl. Metallothionein gene coding sequences were obtained from the GenBank database (A: KF561474.1, B: KF561476.1, and C: KF561475.1) and the primers for the qPCR were designed. The primers for the metallothionein gene quantification were Metallo-A forward: CTGTTGCCAAAACAATTGCTCA; Metallo-A reverse: CTCCAGTGGCACAAATGCAAG; Metallo-B forward: TGCATTCACCACCAGTAGCG; Metallo-B reverse: GCCAAGGTAAATGCTCTCGTGTG; Metallo-C forward: GTGCCCTCGTTGTCAAGGTG; and Metallo-C reverse: ATTGGTTCCACACGTGCAGT. The primers used to amplify L32 were the same as described previously^22^, whose expression was used as a control. Generally, cDNA corresponding to 30 ng of total RNA was used and reaction was performed by using Ex-Taq (Takara Bio Inc., Siga, Japan). Metallothionein gene expressions were normalized to the ribosomal protein L32 level. For the estimation of the gene expression levels, 2^−ΔΔCτ^ method was used (REF:Livac). On completion of the assay, a dissociation stage was carried out for melting curve analysis.

To determine tissue-specific mRNA expression, ten individuals were used for one exposure, and they were soaked in RNA later (Thermo Fisher Scientific, Yokohama, Japan) after the exposure. They were dissected under a stereomicroscope and midgut and hepatopancreas were separated from other tissues. Total RNAs were prepared from the midgut and hepatopancreas and other tissues as described above. Both RNAs were used for qPCR using the same primers and conditions as above.

To evaluate the copy number of the mRNA, ten-fold dilution series of each plasmid DNA containing the target sequence prepared to make the standard curve.

### Generation of transgenic *Daphnia*

To make a reporter gene that can respond to heavy metal exposure, the promoter region of MT-A was fused to the GFP gene. The MT-A gene containing the promoter region and coding sequence was amplified from purified genomic DNA by PCR. *Daphnia* genomic DNA was prepared as described preciously^17^. The primer sequences were TCTCAGTCCAGGTTTGGTTAGGA and TGTCTTAGTTGGGTGCCATTCTC. The amplified DNA fragment was cloned into a pCR-Blunt II-TOPO vector (Thermo Fisher Scientific, Yokohama, Japan), generating pCR-MT-A. After the confirmation of the sequence, to obtain the DNA fragment containing MT-A promoter region and its vector, MT-A open reading frame and the putative three prime untranslated regions (3′-UTR) were removed by PCR using primers GCCCTTGCTCACCATGTTGATTGAAGATTTGGAATAGTGT and TTGTCCAAACTCATCAAGGGCGAATTCCAGCAC. The GFP coding sequence and polyA signal sequence was obtained from pAcGFP-C1 (Clontech Laboratories, Inc., CA, USA) using primers ATGGTGAGCAAGGGCGCCGA and GATGAGTTTGGACAAACCACAACTAGAATGCAGTG. By fusing these two DNA fragments using In-fusion HD Cloning Kit (Clontech Laboratories, Inc., CA, USA), GFP coding sequence and SV40 poly A signal sequence were inserted under the control of MT-A, which was designated as pCR-MTApro-GFP. As a NHEJ targeting site of TALEN, *eyeless*-targeting recognition sites^17^ was used because the site was successfully used in the previous study (REF). To fuse the eyeless site with pCR-MTApro-GFP, the MT-A promoter region with GFP DNA fragment was introduced to the pCS-ey2-4XJHRE-H2B-GFP-ey1, which is the plasmid containing eyeless site and used in the previous study (REF: Nakanishi T., Kato Y., Matsuura T., Watanabe H., (2016) TALEN-mediated knock-in) using the In-fusion Cloning Kit using the primers AAACCTCCACTGAGACTCCGACTGGCGTAATAGCGA and GCGGCCGTTACTAGTCGGTACCCAGCTTTTGTTCC. The chimeric gene was amplified by PCR using primers TCTCAGTGGAGGTTTGGTTAGGA and ACTAGTAACGGCCGCCAGTG. TALEN target sequences, CCGGCGAGAATTCTCGGTCG and ACAACAACACCTTCGGACG were located in exon 6 of eyeless gene. The ey1 TALEN mRNAs targeting these sites were synthesized as described previously^18^ (Supplementary Fig. S1).

Microinjection was performed as described previously^17,23^. Eggs were obtained from two- to three-week-old *Daphnia* directly after ovulation. The eggs were placed in ice-cold M4 medium^24^ containing 80 mM sucrose. The ey1 TALEN mRNA (500 ng/μl) and GFP reporter plasmids (50 ng/μl) were mixed with the injection marker Alexa Fluor 568 dye (Life Technologies Inc., Grand Island, NY, USA) at a final concentration of 0.01 µM. The microinjection was performed on ice and the injected eggs were incubated in a 96-well chamber at 23 °C until hatching.

The transgenic line was screened by the expression of GFP in offspring (Supplementary Fig. S1). GFP expression was observed under a Leica M165C fluorescence stereoscopic microscope (Leica Microsystems Heidelberg GmbH, Mannheim, Germany) fitted with a 480-nm excitation filter and a 510-nm barrier filter (GFP2 filter set). To confirm the transgene, inverse PCR was performed. Approximately 2 μg of genomic DNA was prepared from the transgenic animals, digested with EcoR I, and circularized by ligation using T4 DNA ligase at 4 °C for 24 h. The PCR product using primers derived from the vector sequence (GGGAGAAAGGCGGACAGGTATC and GGTAGCTCTTGATCCGGCAAAC) was sequenced to clarify the junction sequence (Supplementary Fig. S2) after cloning into a pBlunt II-TOPO vector using an In-fusion HD cloning kit (Takara Bio Inc., Siga, Japan).

### Imaging of *D. magna* MetalloG

GFP expression was observed under a Leica M165C fluorescence stereoscopic microscope (Leica Microsystems Heidelberg GmbH, Mannheim, Germany) fitted with a 480-nm excitation filter and a 510-nm barrier filter (GFP2 filter set). Light field and fluorescent images were recorded with a color digital camera (Leica DC500; Leica Microsystems Heidelberg GmbH, Mannheim, Germany) mounted on the microscope. GFP expression was quantified based on the protocol described previously^25^.

To observe endogenous GFP expression of *D. magna* MetalloG, eggs were isolated from brood chamber of *D. magna* MetalloG just after ovulation and placed in 96 well plate containing ADaM medium.

## Results and discussion

### Heavy metal analysis

In order to confirm actual concentrations of heavy metals used for the exposure, each medium was sampled and heavy metals was measured by ICP-MS. ADaM medium, which was used as a control, contained certain amount of Zn (0.1–0.15 µM) in addition to trace amount of Mn, Cu, Cd and Pb (Supplementary Table S1), which were derived from sea salt used for ADaM preparation. ADaM also contains 1.45 µM Se. As mentioned above, these metals may induce GFP expression in a control medium as a background. Estimated concentrations of heavy metals by ICP-MS were generally close to nominal concentrations (Supplementary Table S2).

### Metallothionein gene expression changes with heavy metal exposure

**Figure 1:**
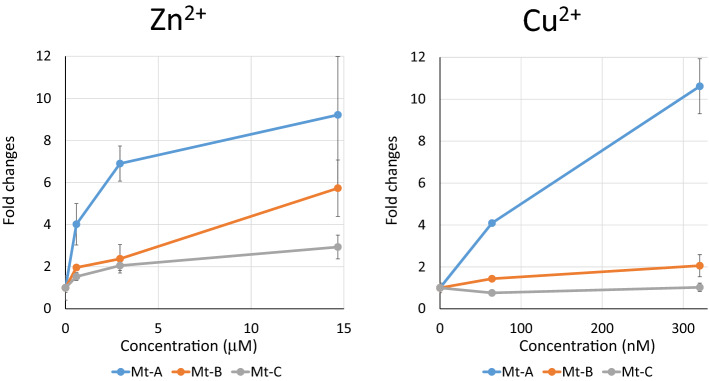
MT gene expression changes in response to heavy metal exposure. Indicated concentrations of Zn^2+^ and Cu^2+^ were exposed to neonate *Daphnia* (< 24 h) for 24 h. Five daphniids were used in 250 mL exposure and total RNA was extracted from the five daphniids. Expression levels of each MT gene were estimated by qPCR, normalized to the L32 level, and divided by the control (unexposed) expression level. The exposure was repeated three times. Bar = SE, *p < 0.05, ^+^*p* ≤ 0.1.

By searching the daphnia genome database (http://arthropods.eugenes.org/EvidentialGene/daphnia/), we identified three MT genes named MT-A, MT-B and MT-C. To select a metallothionein gene that could be effectively and strongly activated by heavy metals, quantitative PCR analysis was performed after exposing daphniids to CuSO_4_. Daphniids were subjected to 64 nM and 320 nM Cu^2+^ for 24 h and the mRNA expression levels of their MT genes were evaluated by q-PCR. MT-A showed the highest response to Cu^2+^ whereas MT-B was only slightly but not significantly activated. MT-C did not respond to Cu^2+^ (Fig. 1). Daphniids were also exposed to different concentrations of ZnCl_2_ for 24 h and the expression levels of MT-A, MT-B, and MT-C were examined. Only MT-A showed > 6× activation in 3 µM and 15 µM Zn^2+^ exposures, and the induction levels were almost the same for both concentrations. MT-B was activated only at the highest Zn^2+^ concentration (15 µM ); however, MT-C was not significantly induced even at that dose (Fig. 1).

MT-A alone showed the highest response to heavy metals. In the present study, MT-B showed weak activation and MT-C hardly respond at all to heavy metals. Our findings are comparable to those of a previous study^12^. The promoter regions of MT-A and MT-B have three metal response element (MRE) motifs and one MRE motif^26,27^, respectively. The presence of MRE in the promoter regions of *Daphnia* MT genes was also mentioned in another study^15^. Therefore, MRE might contribute to metal response and its occurrence in triplicate in MT-A might explain the fact that this metallothionein showed the strongest response to heavy metal exposures.

### Transgenic *Daphnia*

**Figure 2:**
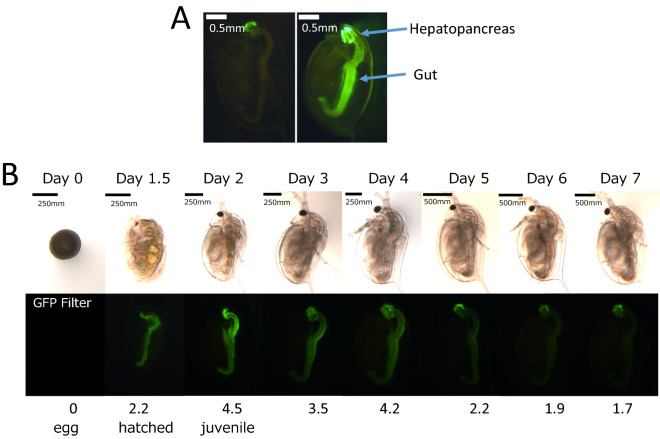
Heavy metal response image of *D. magna* MetalloG and endogenous GFP expression of *D. magna* MetalloG. (**A**) *D. magna* MetalloG daphnia at 7 days old exposed to 30 μM of ZnCl_2_ for 48 h (right panel) and control *D. magna* MetalloG (unexposed) *Daphnia (*left panel) at the same age. (**B**) Eggs were obtained from *D. magna* MetalloG brood chamber after ovulation (assigned as Day 0) and cultured in ADaM. Pictures were taken for one week using the same parameters throughout. Bar = 250 µm. Relative fluorescence intensities were calculated by the method of Törner et al. (23) and indicated under the figures.

We obtained the promoter region and 5′-UTR of MT-A to drive the GFP gene since this metallothionein displayed the strongest response to ZnCl_2_. We obtained 1675 bp upstream sequence from the first ATG of MT-A and used as the MT-A promoter because coding sequence of another gene (Chitinase) was located further upstream region. We included a recognition sequence of *eyeless*-targeting TALEN named ey1 TALEN as previously constructed^18^ into the donor DNA (Supplementary Fig. S1), because we previously reported that *Daphnia magna eyeless* (*Dma-ey*, ortholog of mammalian *pax6*) gene can be used as a target site for recombination. This plasmid was knocked-in via TALEN-mediated non-homologous end-joining repair as described previously^18^. We injected 374 eggs and isolated one transgenic line designated as *D. magna MetalloG* (Fig. 2A, Supplementary Fig. S1). Transmission of the GFP reporter gene was confirmed by PCR using the progeny of the line and the integration site was sequenced. The transgenic *Daphnia* is suppsed to have 6 kb (3 kb of reporter gene and 3 kb of its vector) DNA fragment as a transgene. The junction sequence is shown in the Supporting Information (Supplementary Fig. S2).

### Endogenous expression of GFP

*D. magna* MetalloG was cultured in ADaM and endogenous GFP expression was observed. During embryogenesis, GFP fluorescence could not be detected (data not shown). After hatching, GFP fluorescence was detected in the midgut and hepatopancreas (Fig. 2B day 1.5). The GFP fluorescence intensity in these organs was highest at day 2, then gradually decreased. At days 6 and 7, fluorescence in the midgut almost disappeared, and weak GFP expression could only be detected in the hepatopancreas (Fig. 2B). When the metal-free medium was used (M4 medium^24^ without any heavy metals), GFP expression could not be detected (data not shown). This result suggests that GFP expression observed at day 2 in ADaM medium was induced by the presence of metals contained in the ADaM medium itself (Supplementary Table S1). To develop a higher S/N ratio of *D. magna* MetalloG, we attempted to use the metal-free medium, however the *D. magna* MetalloG could not survive in the medium, suggesting the importance of MT-A expression or presence of heavy metals during early stages.

### Tissue specific expression of MT-A and GFP

**Figure 3:**
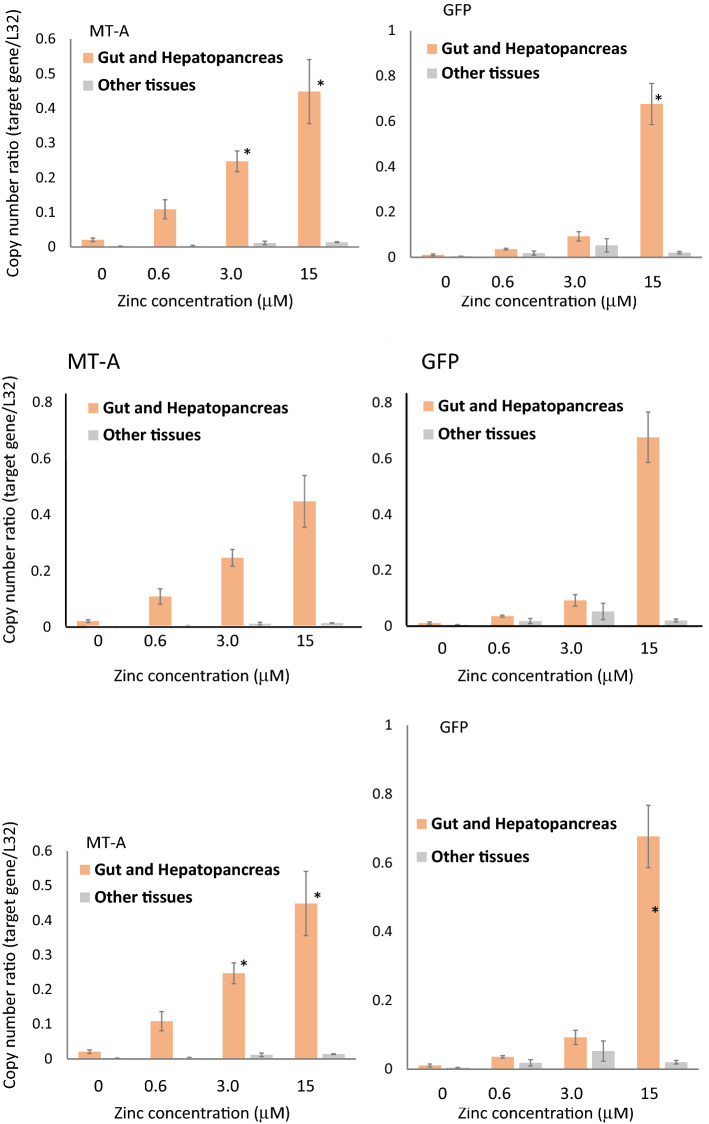
Tissue-specific MT-A and GFP expressions. Neonatal *Daphnia* (< 24 h) were exposed to different concentrations of Zn^2+^ for 24 h. Midgut and hepatopancreas of *D. magna* MetalloG were dissected and mRNA was purified. mRNA was also purified from the remaining tissue. The mRNA expression levels of MT-A and GFP were estimated by qPCR. Relative amounts of mRNA to that of the ribosomal L32 protein gene were estimated by ΔΔCτ method and indicated in Y-axis. The quantification was performed three times. Bar = SE.

To compare the expression patterns of endogenous MT-A and GFP, *D. magna* MetalloG were dissected and their midgut and hepatopancreas and other tissues were separated after exposure to various concentrations of Zn^2+^. As shown in Fig. 3, the expression of both MT-A mRNA and GFP mRNA was exclusively observed in midgut and hepatopancreas and there was little expression in other tissues. They increased to a similar extent in a dose-dependent manner in the midgut and hepatopancreas. Therefore, the MT-A promoter used to drive GFP reflects endogenous MT-A expression well.

Although in situ hybridization is necessary to precisely compare the specific expression of endogenous MT-A and GFP transgene, our comparison of mRNA expression levels in the midgut and in other *D. magna* MetalloGtissues supports the fact that GFP expression mimics that of MT-A. This suggests that the promoter used in the present study contained both metal response element and DNA elements essential for tissue-specific expression in the hepatopancreas and midgut. Since these tissues are continuously exposed to the external environment, it is reasonable that the MT gene is expressed to protect cells from heavy metals existing in the external environment. It is notable that MT-A mRNA and GFP mRNA expression were comparable to the expression level of ribosomal L32 protein mRNA (Supplementary Table S3). As ribosomal proteins are ones of proteins highly expressed in cells, this result suggests the presence of abundant MT mRNA and probably MT protein in these tissues. When mRNA was prepared, a nearly tenfold induction of MT-A mRNA was observed (Fig. 1) and a greater than 20-fold induction was observed in the isolated tissues. This higher induction of mRNA facilitates the induction and detection of GFP. Heavy metal accumulation in the hepatopancreas has been reported in crayfish^28^ and heavy metal MT induction in the same organ was indicated in crabs^29^, which suggests that the hepatopancreas sequesters and detoxifies heavy metals in crustaceans. GFP expression of the hepatopancreas in *D. magna* MetalloG suggests that hepatopancreas function is widely conserved in crustacean including *Daphnia* and tissue functions in this organism.

### GFP expression by single heavy metal exposure

**Figure 4:**
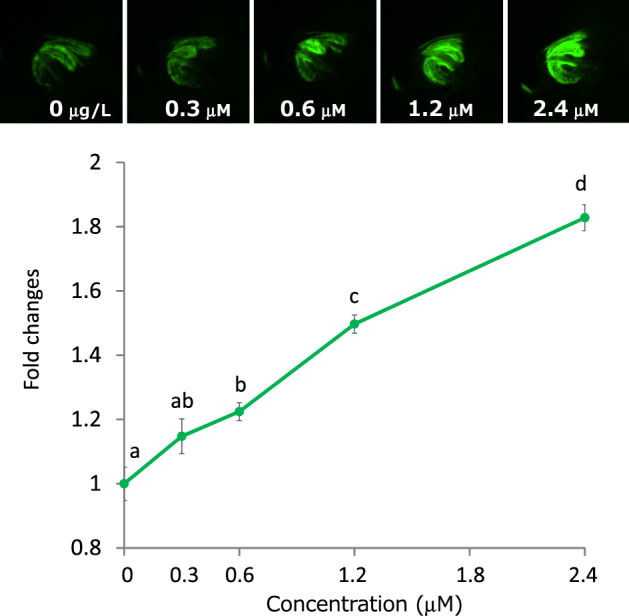
GFP expression changes after ZnCl_2_ exposure for 1 h. Top: representative images of hepatopancreas exposed to ZnCl_2_ for 1 h. Bottom: GFP expressions were quantified and fold changes vs. 0 mg/L fluorescence intensity were calculated. Five daphniids were exposed in 10 mL of ADaM for 1 h. Fold changes vs. unexposed control were plotted. N = 5; **p < 0.01 (t-test).

Based on endogenous GFP expression, seven-day-old *Daphnia* was used in heavy metal exposure tests when the background GFP levels were at a minimum (Fig. 2B day 7). When *D. magna* MetalloG was exposed to Zn^2+^ for 1 h, the GFP expression in the hepatopancreas increased. GFP expression was significantly enhanced at Zn^2+^ concentrations 1.2 µM (Fig. 4) and GFP intensity increased with Zn^2+^ concentration. These results were consistent with those of the MT-A gene activation test (Fig. 1).

**Figure 5:**
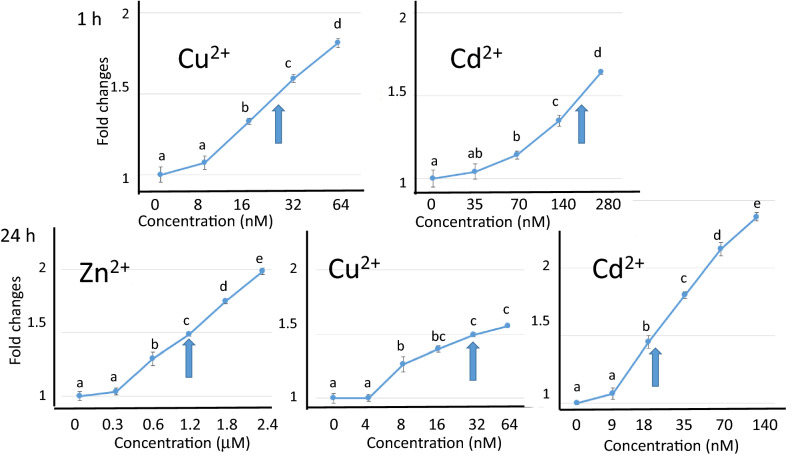
Dose depenent GFP expression changes after heavy metal exposure for 1 h or 24 h. Five daphniids were exposed in 10 mL of ADaM containing indicated metal ions for 1 h or 24 h. GFP expression was quantified and normalized to the control (0 µg/L) expression level. Numbers on x-axis indicate concentrations of each heavy metal (µM for Zn^2+^ and nM for Cu^2+^ and Cd^2+^). Y-axis indicates fold changes. Arrows indicate EC_IR1.5_ values. Top: 1 h exposure, Bottom: 24 h exposure. N = 5; ***p < 0.001; **p < 0.01; *p < 0.05 (t-test).

The responses of *D. magna* MetalloG to Cu^2+^ and Cd^2+^ were also examined after 1 h exposure. As shown in Fig. 5, GFP expression was enhanced in a dose-dependent manner in the presence of either Cu^2+^ or Cd^2+^. The minimum concentrations of Cu^2+^ and Cd^2+^ that significantly enhanced GFP expression were 130 nM and 70 nM, respectively (Fig. 5, Supplementary Table S4).

To determine whether longer exposures to heavy metals reduced detection levels, we examined GFP expression changes after 24 h. GFP expression increased with metal exposure time (Fig. 5). Significant enhancements of GFP expression were observed with 0.6 µM ZnCl_2_, 67 nM Cu^2+^, and 18 nM of Cd^2+^. These values did not change even after 48 h exposure (Supplementary Table S4) and longer (Supplementary Fig. S3). These values were much lower than EC50 values of conventional chronic toxicity test for 24 h*.* When EC50 values were determined using wild-type, they were as follows: Zn^2+^: 180 µM, Cu^2+^: 0.43 µM, and Cd^2+^: 1.7 µM (Supplementary Table S4). Therefore, the GFP response in transgenic *Daphnia* might prove useful in the detection of heavy metals.

### GFP expression by multiple heavy metals exposure

**Figure 6:**
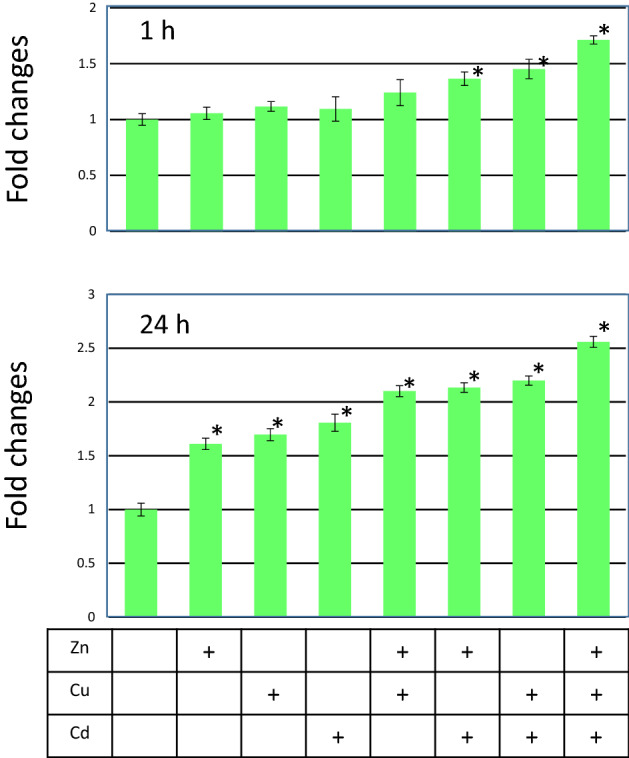
GFP expression by multiple heavy metals exposure. Seven day old *D. magna* MetalloG was exposed to 0.9 µM of Zn^2+^, 20 nM of Cu^2+^, or 110 nM of Cd^2+^, and a combination of these metals. After 1 h and 24 h exposure, GFP expression was quantified, These values were divided by that of control (0 µg/L) expression level and fold changes were calculated. Metal ions used for the exposure are indicated in the boxes (+presence of ion indicated on the left column (Y-axis indicates fold changes. *p < 0.05 (t-test) Measured concentrations are indicated in Supplementary Table S5.

To examine whether *D. magna* MetalloG responded to multiple metal exposure, *D. magna* MetalloG was exposed to 0.9 µM of Zn^2+^, 20 nM of Cu, or 110 nM of Cd^2+^, and a combination of these metals. As shown in Fig. 6, when Cd^2+^ was combined with other metals, GFP fluorescence was significantly induced at 1 h. GFP fluorescence was the highest when the three metals were mixed and exposed. Although there was no significance in the exposure of Zn^2+^ and Cu^2+^, GFP was also induced. These results indicated that GFP fluorescence could be induced additively when multiple heavy metals were exposed. After 24 h exposure, each metal induced GFP expression and additive effect of the mixture of metals could be prominently observed. Only one dose of the three metals were applied in the present study, additional experiments using various concentrations of metals would further clarify the GFP induction profile. Measured concentrations of the medium are indicated in Supplementary Table S5, which shows measured concentrations were close to nominal concentrations.

### Advantages of *D. magna* MetalloG

The use of GFP has been proposed previously^6^ and applied as a reporter gene in other species. To detect heavy metals in the water, heavy metal monitoring medaka have been reported. The GFP in the transgenic medaka is driven by Hsp70, which responds to 110 nM Cd^2+^ after 24 h exposure^30^. *D. magna* MetalloG can respond to 18 nM Cd^2+^ after 24 h; therefore, the *Daphnia* biosensor is more sensitive than the medaka. In addition, Hsp70 can respond not only to metals but to other stimuli including heat; therefore, the expression of GFP does not necessarily indicate the presence of heavy metals. Conversely, the metallothionein gene responds to particular metals and could, therefore, be useful for the classification of toxicants. Recently, transgenic zebrafish containing the GFP gene under the metallothionein promoter have been developed; however, the transgenic zabrafish only responded to high metal concentrations (Cd^2+^: 0.5 ppm (4.5 µM), Zn^2+^: 20 ppm (306 µM);, and Cu^2+^: 0.3 ppm (4.7 µM))^31^. While the promoter used for the detection of heavy metal was MT, sensitivity to metals was more than hundred times higher in *Daphnia*. To detect heavy metals in soil, a transgenic nematode was also established^32^. In the transgenic nematode, the lowest observed effect concentration to Cd^2+^ was reported as 5 µM (0.9 ppm, 900 µg/L). These results indicate that *Daphnia* is a suitable species for the detection of heavy metals in much lower concentrations, which might be reasonable because *Daphnia* is known to be more sensitive to heavy metals than fish. Therefore, *D. magna* MetalloG is the most sensitive and rapid GFP-expressing biosensor ever developed.

Compared to zebrafish and medaka, *Daphnia* has application advantages. *Daphnia* is an invertebrate; therefore, there are no legal ethical issues with this genus during experimental studies. As the regulation of animal use is becoming stricter, the application of invertebrates is preferable to vertebrates. In addition, *Daphnia* can mature in one week and start reproduction, which facilitates propagation and use. Based on genome editing techniques in *Daphnia* developed in our laboratory, this is the first reported study of a transgenic strain utilized as a biosensor strain. Our technique provides several types of biosensors using *Daphnia* in the near future.

### Perspective of the use of *D. magna* MetalloG

*D. magna* MetalloG still has problems that need to be improved. One is endogenous expression and the other is tissue specific expression. GFP expression was detected from Daphnia’s exposure to the ADaM, which contained trace amounts of Mn, Rb, Sr, Co, Se, and other metals (Supplementary Table S1). To eliminate these heavy metal ions, we examined if metal free synthetic medium could be used for the test. When the synthetic water was used (M4 medium without any metal ions), the endogenous GFP expression was significantly reduced; however, the daphniids died. By reducing the amounts of metals in medium to minimum levels, it might be possible to decrease the lower limit of sensitivity. Exclusive GFP expression might not be desirable as a biosensor because observation of *Daphnia* with high magnification is required. If the *Daphnia* expresses GFP in their entire body, it becomes much easier to detect the expression, possibly without using a microscope. Therefore, the rational design of the promoter by eliminating the DNA element responsible for tissue-specificity and, adding multiple MER might help further development of *Daphnia* as a biosensor.

The detection limits to these heavy metals of *D. magna* MetalloGnking Water Regulations, USEPA^33^ Inorganic Maximum Contaminant Level Goal of Copper and Cadmium were assigned as 1.3 mg/L and 0.005 mg/L, respectively. Secondary Maximal Contaminant Level of Zinc was assigned as 5 mg/L in Secondary Drinking Water Standards, USEPA^34^. Thus, *D. magna* MetalloG might be useful for detecting heavy metals near regulatory levels.

In conclusion, we developed a genome-edited *Daphnia* that can express GFP that responds to heavy metals. The sensitivity of GFP expression is much higher than that of conventional acute toxicity testing. In addition, genome-edited *Daphnia* can detect heavy metals much faster (1 h) than the conventional method (24–48 h). *Daphnia* is particularly sensitive to aquatic chemical stresses. By introducing a sensitive reporter gene into a susceptible organism, a highly accurate bioassay could be created. The introduction of other reporter genes responsive to other toxicants can build genome-edited *Daphnia* that facilitate water quality monitoring and contaminant identification.

## Supplementary information

Supplementary Legends.

Supplementary Information.
