# Supplementary material for: Production of genome-edited Daphnia for heavy metal detection by fluorescence
Source: Sci Rep. 2020 Dec 8;10:21490. doi: 10.1038/s41598-020-78572-z (PMC7722880; doi:10.1038/s41598-020-78572-z)
Supplement: Supplementary file 1 — Supplementary Legends. [file 41598_2020_78572_MOESM1_ESM.docx]

Production of genome edited *Daphnia* for heavy metal detection by fluorescence

Takuto Arao^1^, Yasuhiko Kato^1,2^, Quang Dang Nong^1^, Hiroshi Yamamoto^3^, Haruna Watanabe^3^, Tomoaki Matsuura^1^, Norihisa Tatarazako^4^, Akira Okamoto^5^, Takeru Matsumoto^5^, Hajime Watanabe^1^

1 Department of Biotechnology, Graduate School of Engineering, Osaka University, Suita, Osaka, Japan

2 Frontier Research Base of Global Young Researchers, Graduate School of Engineering, Osaka University, Suita, Japan

3 Center for Health and Environmental Risk Research, National Institute for Environmental Studies, Tsukuba, Japan

4 Faculty of Agriculture, Ehime University, Matsuyama, Ehime, Japan

5 Department of Environmental Science & Toxicology, Nippon Soda Co., Ltd., Kanagawa, Japan

* Corresponding author

E-mail:watanabe@bio.eng.osaka-u.ac.jp

**Supplemental Information**

Figure S1

Schematic diagram for the construction of reporter plasmid and structure of the integrated gene. Orientations of primers are indicated by red arrows. Names of plasmids used in the text are also indicated.

Figure S2

Nucleotide sequence of the integration site of *D. magna* MetalloG. Using *D. magna* MetalloG genomic DNA, inverse PCR was performed and the nucleotide sequence of the integration site was determined. Upper sequence shows wild type allele of eyeless gene at the integration site and TALEN target sites are indicated in red..Lower sequence shows the junction sequence. Nucleotide sequence with gray background shows wild type sequence. Nucleotide sequence in green shows integrated DNA sequence originated from pBR322. Unidentifiede sequcence which may be the result of DNA repair is indicated in gray letters.

The other integration site could not be clarified because of an unknown reason.

Figure S3

GFP expression of *D. magna* MatalloG after longer ZnCl_2_ exposure

*D. magna* MetalloG was exposed in 50 mL of dechlorinated tap water containing ZnCl_2_ for 24, 48, 72 and 96 h_._ The concentrations ( μg/L) are indicated on the left.

Table S1

Metal concentrations of ADaM medium measured by ICP-MS

Metal concentrations of the medium used as a control were measured by ICP-MS. Measurement was repeated three times and average values are indicated. Carrier gasses for the analyses are indicated in the parenthesis.

Table S2

Metal concentrations of single metal exposure medium measured by ICP-MS. Various concentrations of Zn, Cu or Cd was dissolved to ADaM medium and daphniids were exposed for 24 h. At 0 h, 1 h, 2 h and 24 h, samples were obtained and metals were measured by ICP-MS. Measured ions are indicated on the left (-: ADaM only). Nominal concentrations (s.econd column from the left) and measured concentrations at each time point are indicated. Measurement was repeated three times and average values are indicated.

Table S3

mRNA expression of MT-A and GFP in hepatopancreas.

Neonatal *D. magna* MetalloG (< 24 h) were exposed to different concentrations of ZnCl_2_ for 24 h. Midgut and hepatopancreas of *D. magna* MetalloG were dissected and mRNA was purified. The mRNA expression level of Matallo A andGFP were estimated by qPCR and copy numbers were calculated using control plasmid DNAs pCR-MT-A and pCS-ey2-MTApro-GFP-ey1, respectively. Primers for GPF mRNA quantification were TACGGCAAGCTGACCCTGAA and CTGCTGCCCGATAACCACTAC. Names of the amplified genes, concentrations of ZnCl_2_ are indicted in the Well Name.

Table S4

Detection limits of *D. magna* MetalloG

Exposure times (1, 2, 24, 48 h) and the lowest significant concentrations (µM ) are indicated. To compare these values with conventional method, EC50s in a previous study (REF) are also indicated.

Table S5

Metal concentrations of multiple metal exposure medium measured by ICP-MS.

0.9 µM of Zn^2+^, 20 nM of Cu^2+^, or 110 nM of Cd^2+^ or combinations of these ions were dissolved to ADaM medium and daphniids were exposed for 24 h. At 0 h, 1 h and 24 h, samples were obtained and metals were measured by ICP-MS. Measurement was repeated three times. Ions used for the exposure are indicated on the left column. At each time point, measured concentrations are indicated each column with ion names. NS: not detected.
