# Supplementary material for: Production of genome-edited Daphnia for heavy metal detection by fluorescence
Source: Sci Rep. 2020 Dec 8;10:21490. doi: 10.1038/s41598-020-78572-z (PMC7722880; doi:10.1038/s41598-020-78572-z)
Supplement: Supplementary file 2 — Supplementary Information. [file 41598_2020_78572_MOESM2_ESM.pptx]

## Slide 1
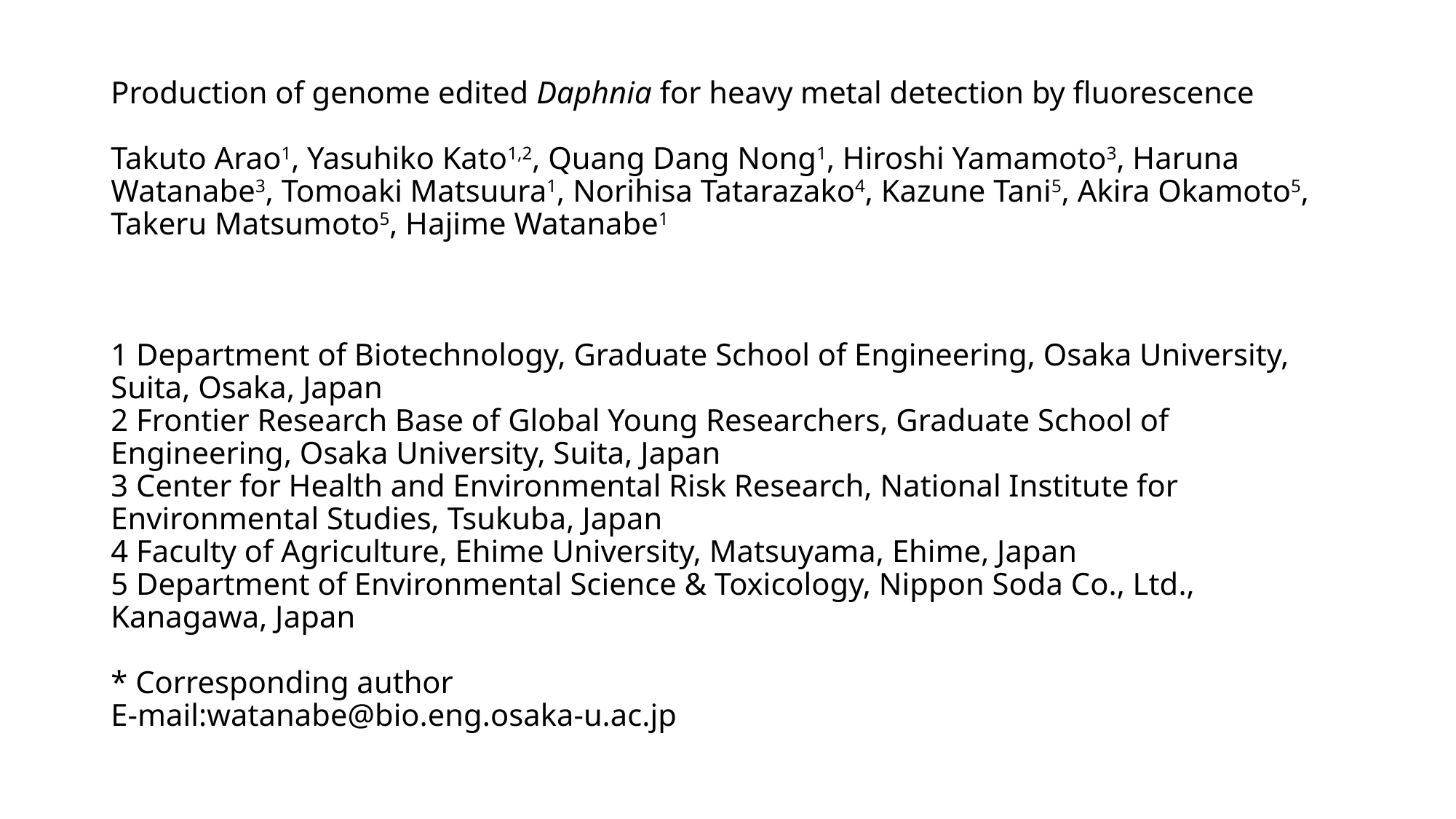

# Production of genome edited Daphnia for heavy metal detection by fluorescence   Takuto Arao1, Yasuhiko Kato1,2, Quang Dang Nong1, Hiroshi Yamamoto3, Haruna Watanabe3, Tomoaki Matsuura1, Norihisa Tatarazako4, Kazune Tani5, Akira Okamoto5, Takeru Matsumoto5, Hajime Watanabe1       1 Department of Biotechnology, Graduate School of Engineering, Osaka University, Suita, Osaka, Japan 2 Frontier Research Base of Global Young Researchers, Graduate School of Engineering, Osaka University, Suita, Japan 3 Center for Health and Environmental Risk Research, National Institute for Environmental Studies, Tsukuba, Japan 4 Faculty of Agriculture, Ehime University, Matsuyama, Ehime, Japan 5 Department of Environmental Science & Toxicology, Nippon Soda Co., Ltd., Kanagawa, Japan   * Corresponding author E-mail:watanabe@bio.eng.osaka-u.ac.jp

## Slide 2
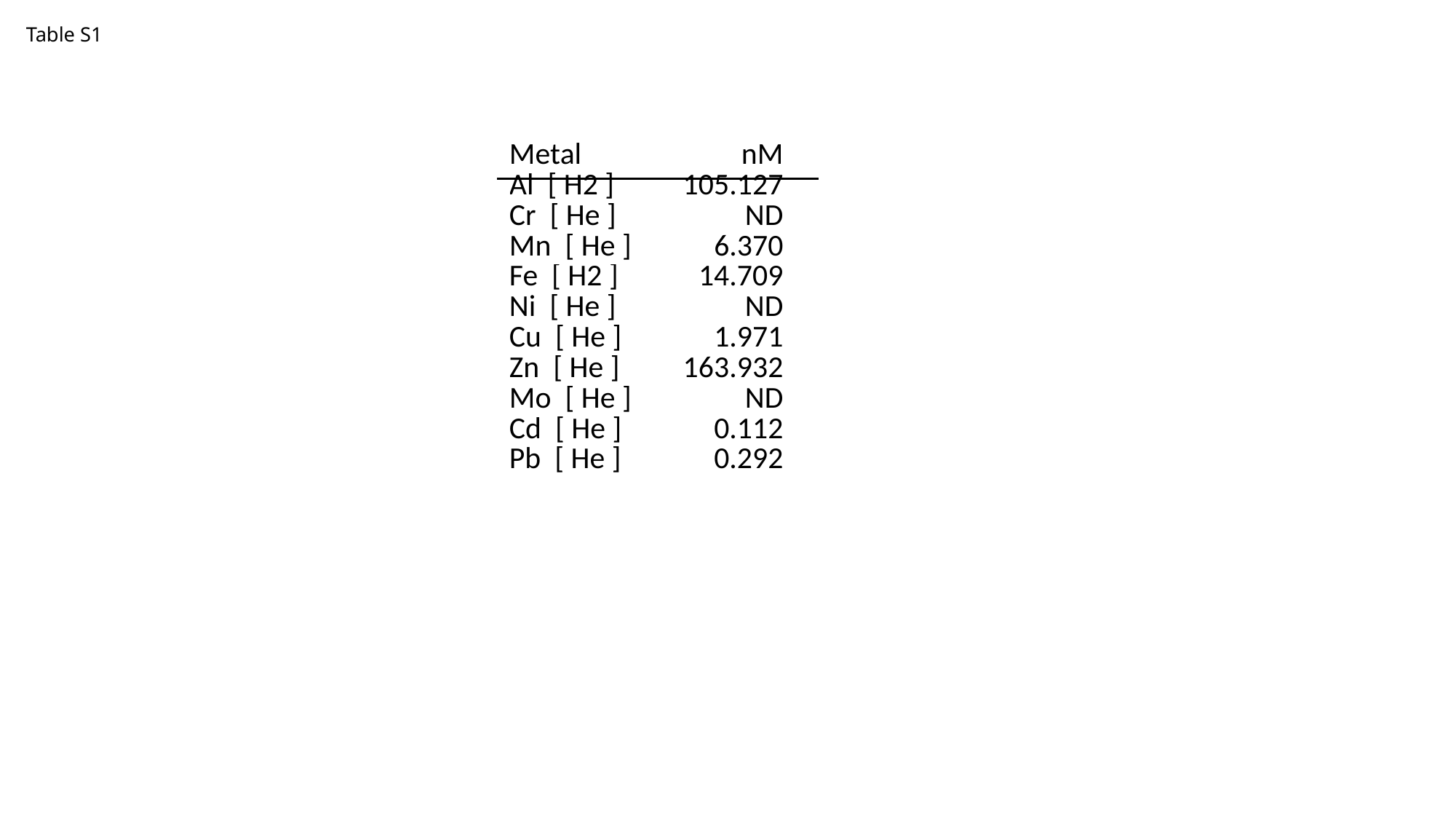

# Table S1
| Metal | nM | RSD |
| --- | --- | --- |
| Al [ H2 ] | 105.127 | 3.800 |
| Cr [ He ] | ND | 15.638 |
| Mn [ He ] | 6.370 | 1.394 |
| Fe [ H2 ] | 14.709 | 8.028 |
| Ni [ He ] | ND | N/A |
| Cu [ He ] | 1.971 | 24.308 |
| Zn [ He ] | 163.932 | 2.619 |
| Mo [ He ] | ND | 10.249 |
| Cd [ He ] | 0.112 | 65.718 |
| Pb [ He ] | 0.292 | 6.779 |

## Slide 3
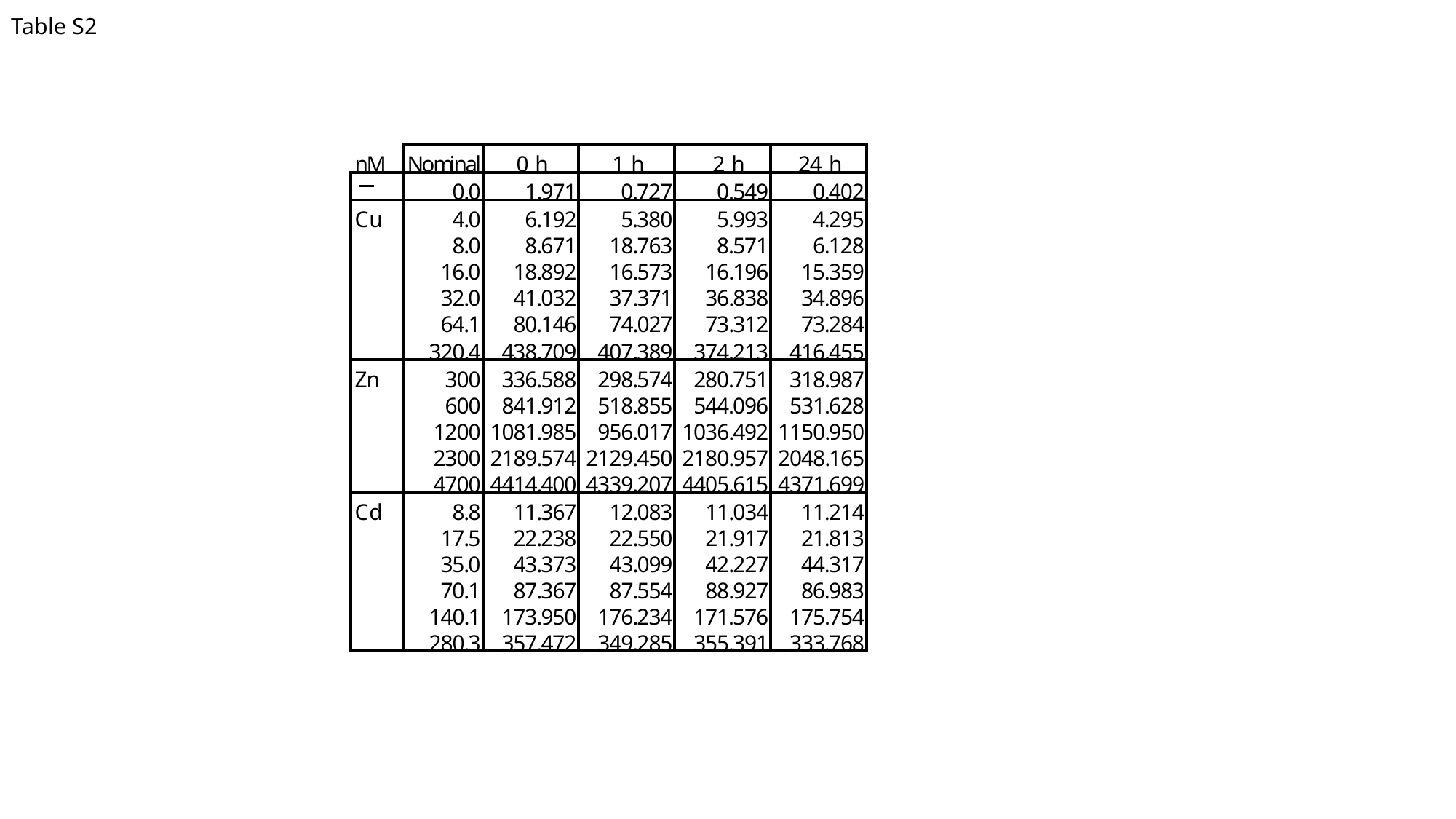

# Table S2

## Slide 4
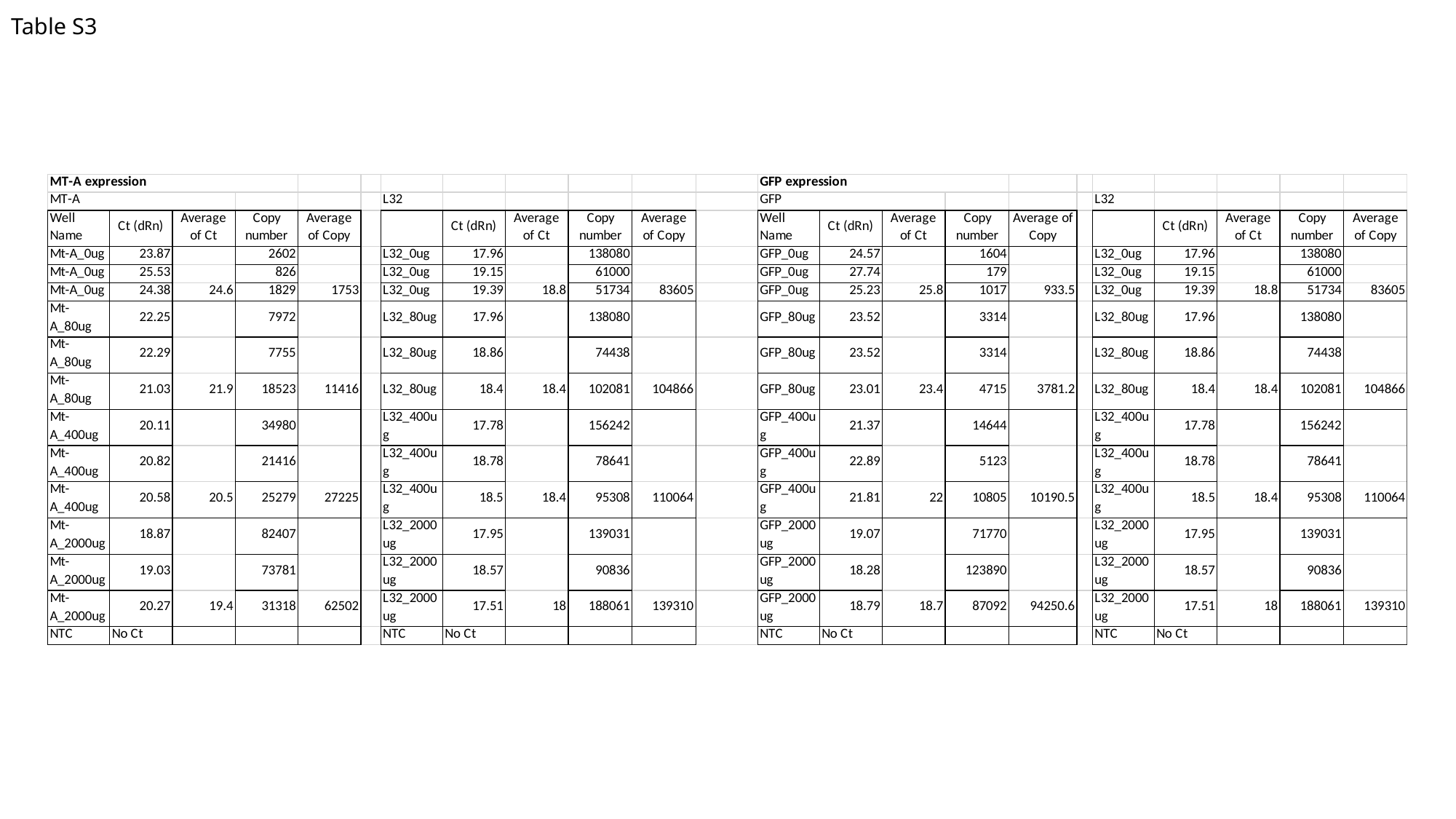

# Table S3

## Slide 5
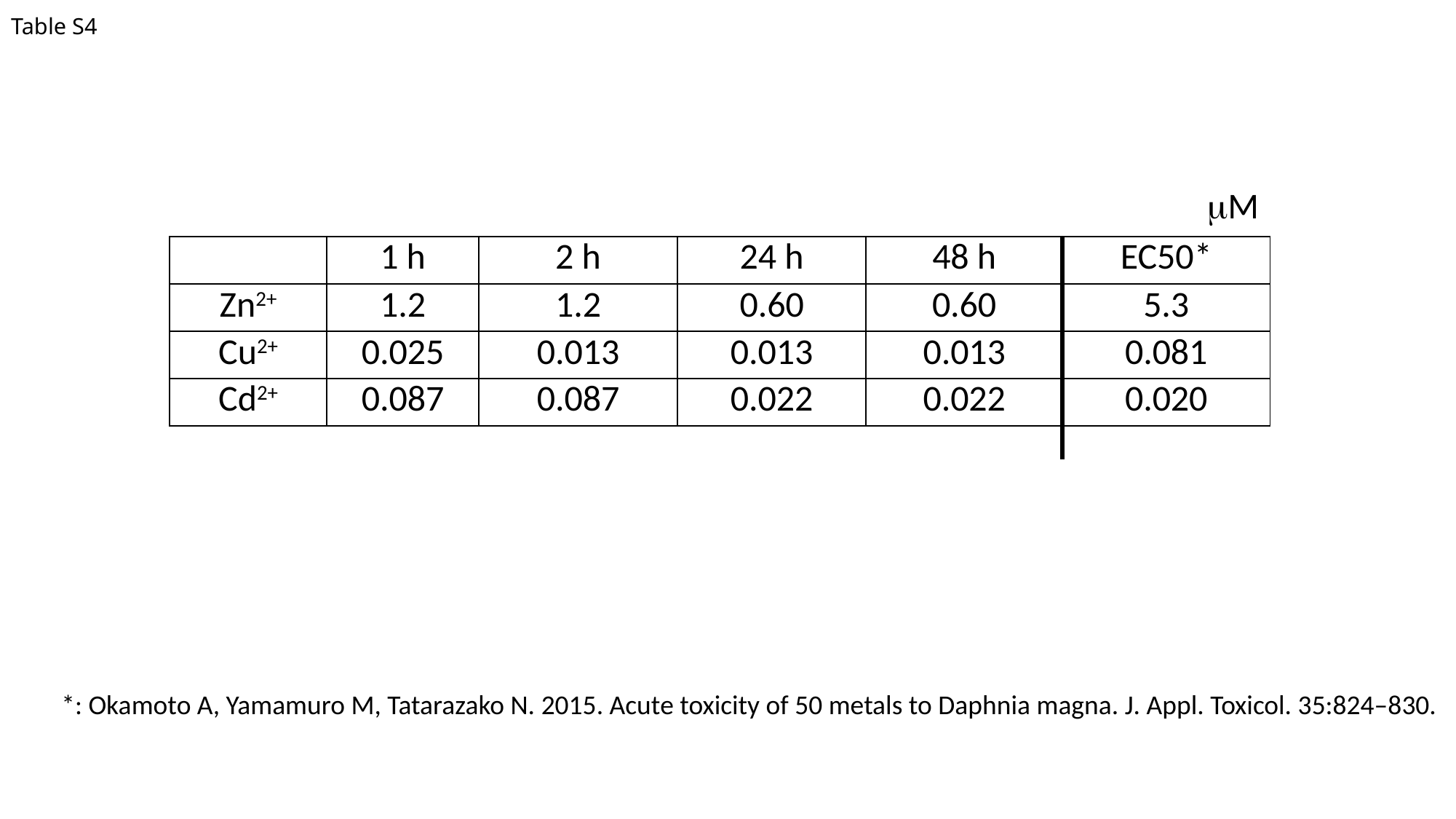

# Table S4
mM
| | 1 h | 2 h | 24 h | 48 h | EC50\* |
| --- | --- | --- | --- | --- | --- |
| Zn2+ | 1.2 | 1.2 | 0.60 | 0.60 | 5.3 |
| Cu2+ | 0.025 | 0.013 | 0.013 | 0.013 | 0.081 |
| Cd2+ | 0.087 | 0.087 | 0.022 | 0.022 | 0.020 |
*: Okamoto A, Yamamuro M, Tatarazako N. 2015. Acute toxicity of 50 metals to Daphnia magna. J. Appl. Toxicol. 35:824–830.

## Slide 6
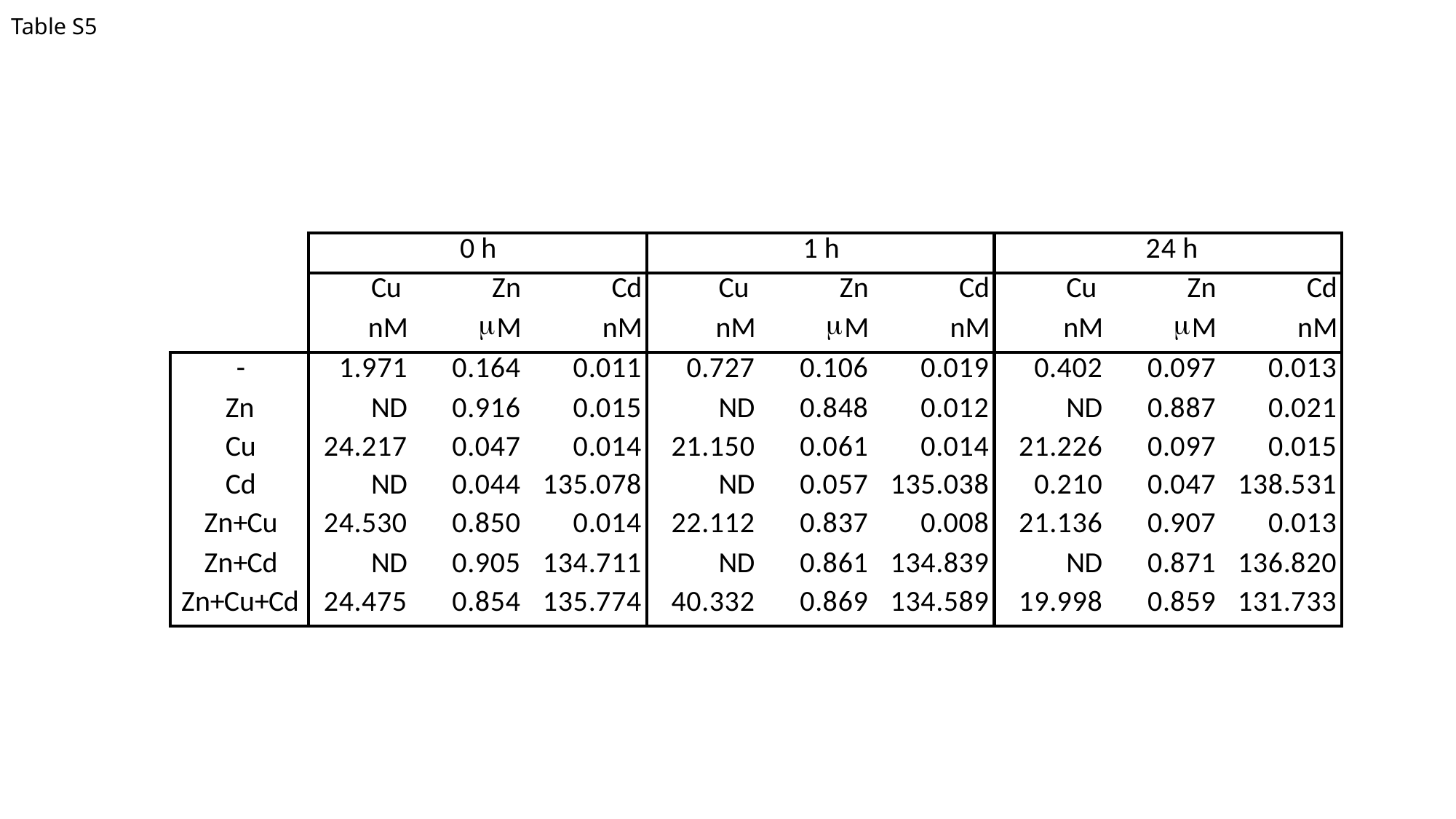

# Table S5

## Slide 7
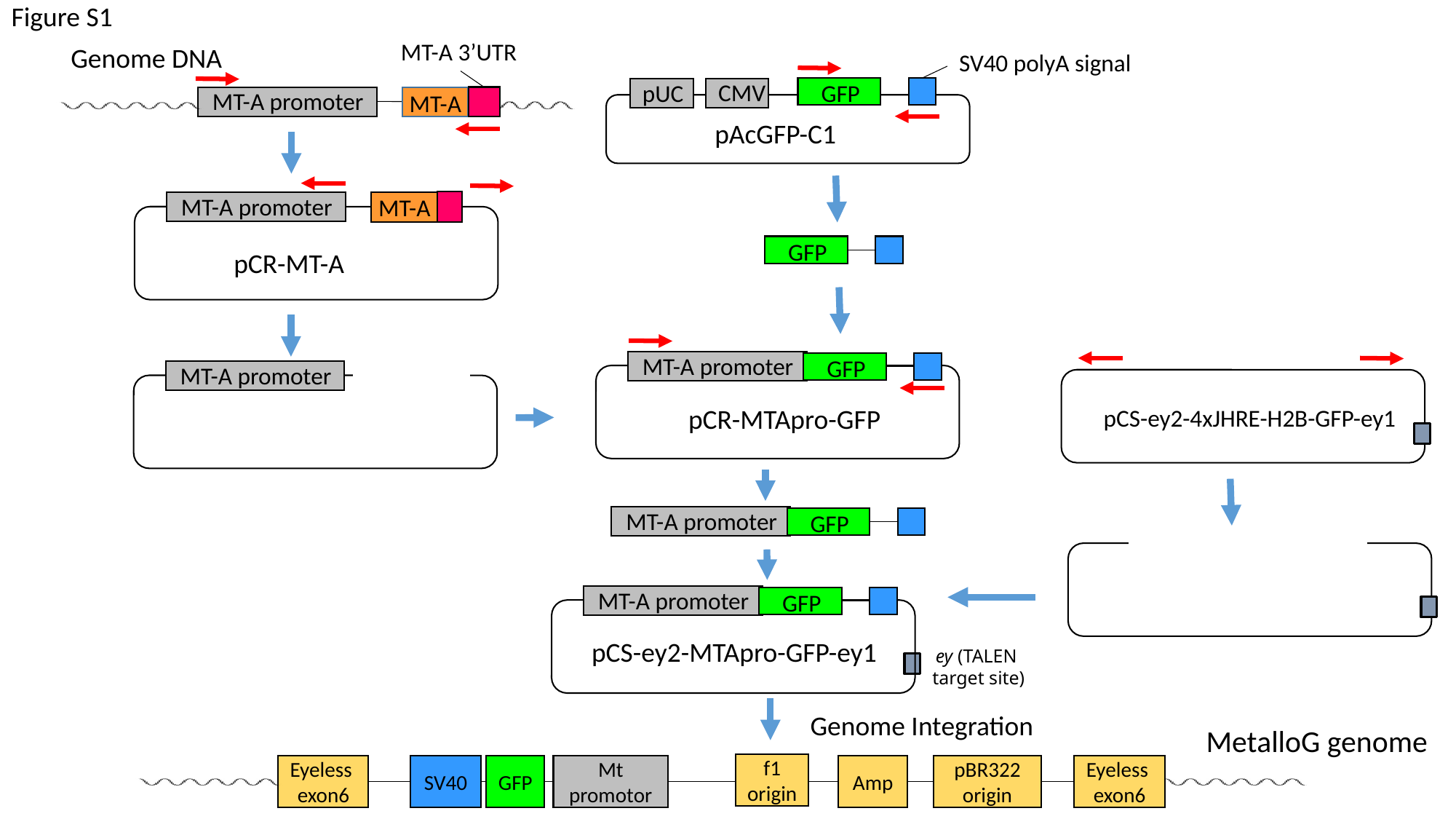

Figure S1
MT-A 3’UTR
Genome DNA
SV40 polyA signal
CMV
pUC
GFP
MT-A promoter
MT-A
pAcGFP-C1
MT-A promoter
MT-A
GFP
pCR-MT-A
MT-A promoter
GFP
MT-A promoter
MT-A
pCR-MTApro-GFP
pCS-ey2-4xJHRE-H2B-GFP-ey1
MT-A promoter
GFP
MT-A promoter
GFP
pCS-ey2-MTApro-GFP-ey1
ey (TALEN
target site)
Genome Integration
MetalloG genome
f1 origin
Eyeless
exon6
SV40
GFP
Mt promotor
Amp
pBR322
origin
Eyeless
exon6

## Slide 8
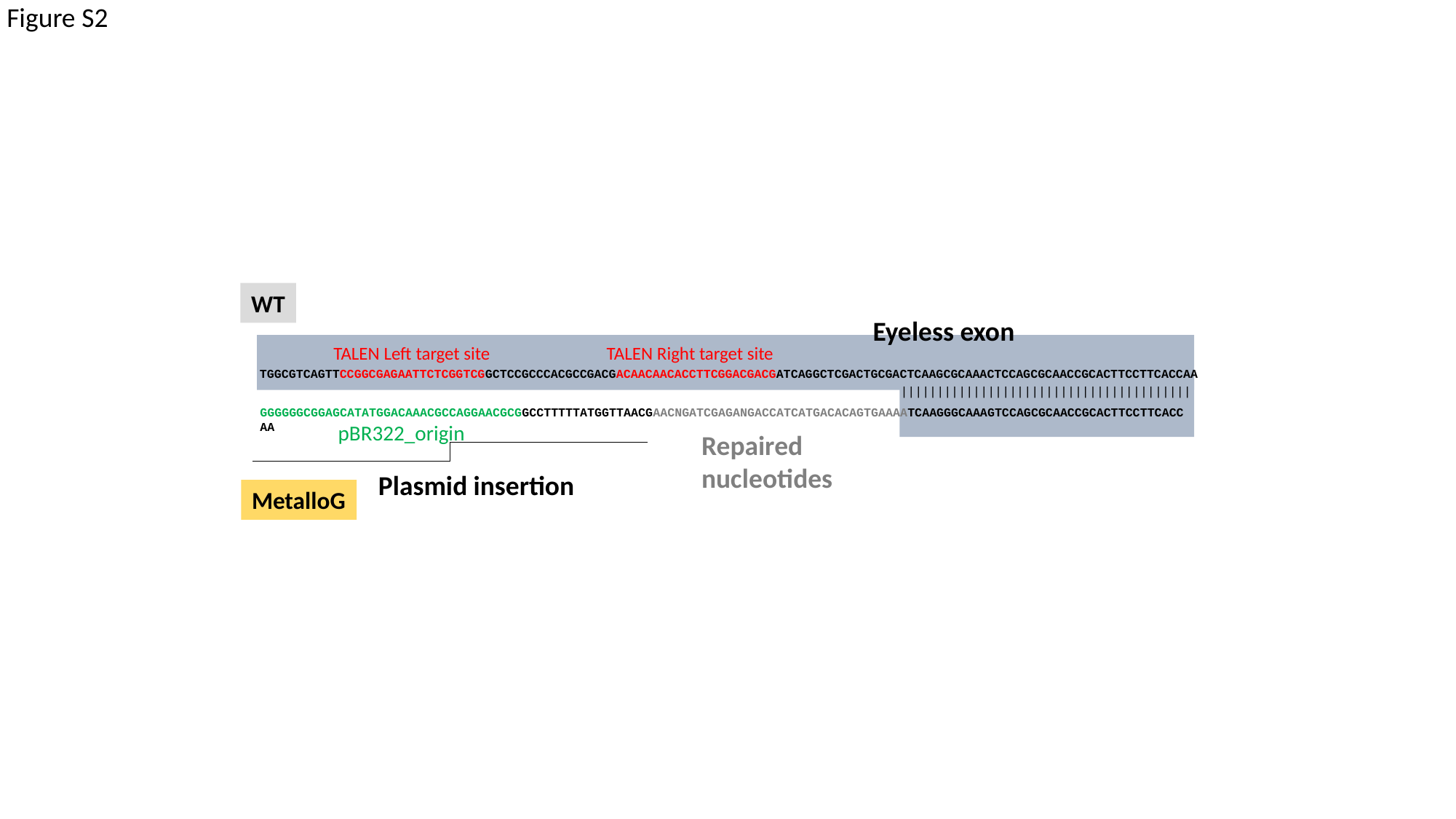

Figure S2
WT
Eyeless exon
TALEN Left target site
TALEN Right target site
TGGCGTCAGTTCCGGCGAGAATTCTCGGTCGGCTCCGCCCACGCCGACGACAACAACACCTTCGGACGACGATCAGGCTCGACTGCGACTCAAGCGCAAACTCCAGCGCAACCGCACTTCCTTCACCAA
||||||||||||||||||||||||||||||||||||||||
GGGGGGCGGAGCATATGGACAAACGCCAGGAACGCGGCCTTTTTATGGTTAACGAACNGATCGAGANGACCATCATGACACAGTGAAAATCAAGGGCAAAGTCCAGCGCAACCGCACTTCCTTCACCAA
pBR322_origin
Repaired
nucleotides
Plasmid insertion
MetalloG

## Slide 9
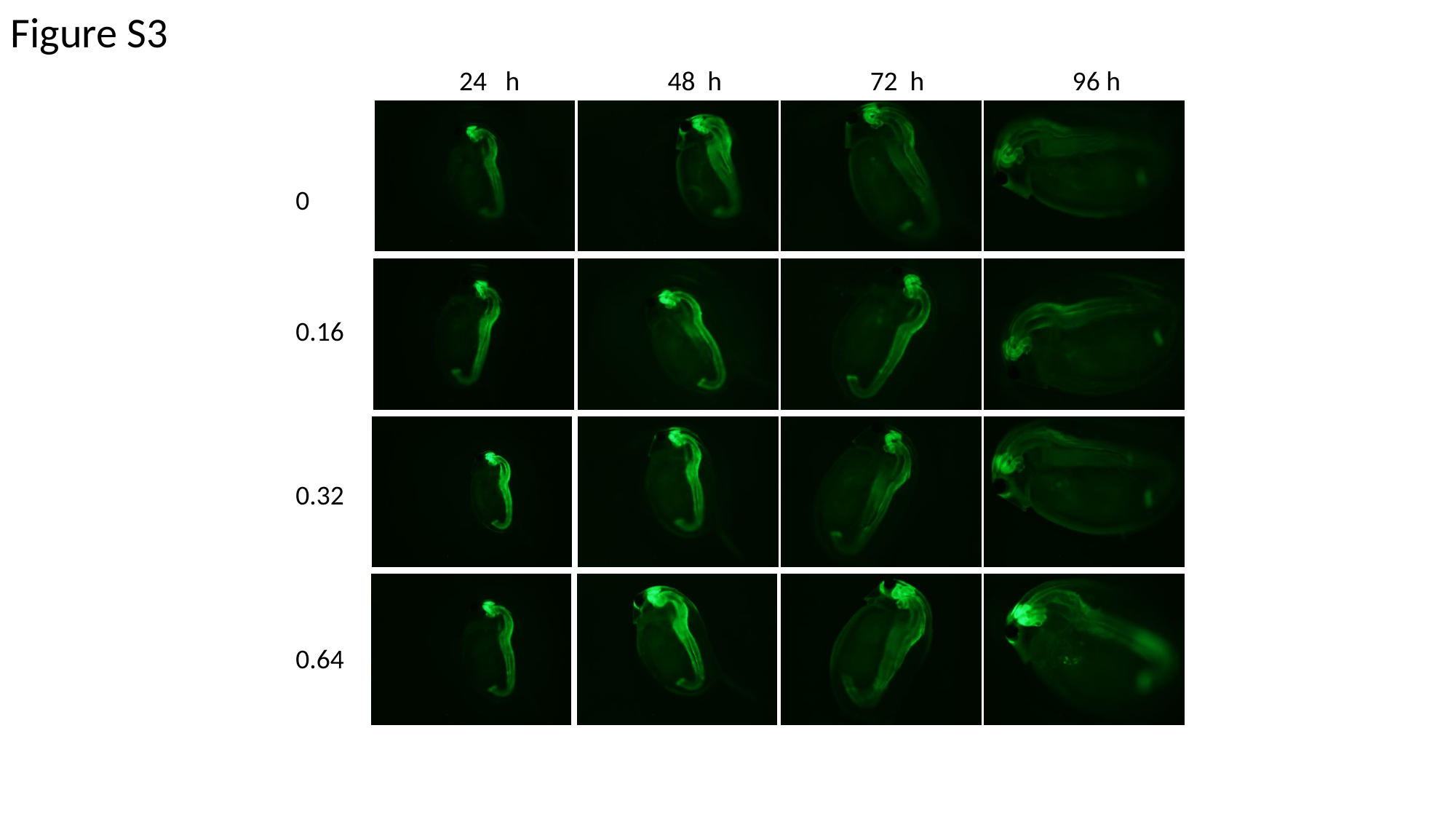

Figure S3
24 h 48 h 72 h 96 h
0
0.16
0.32
0.64
